# Bimetallic Au–Ag Nanoparticles: Advanced Nanotechnology for Tackling Antimicrobial Resistance

**DOI:** 10.3390/molecules27207059

**Published:** 2022-10-19

**Authors:** Chandrashekhar Singh, Abhishesh Kumar Mehata, Vishnu Priya, Ankit Kumar Malik, Aseem Setia, M. Nikitha Lakshmi Suseela, Vikas, Patharaj Gokul, Samridhi, Sanjeev K. Singh, Madaswamy S. Muthu

**Affiliations:** 1 Department of Pharmaceutical Engineering & Technology, Indian Institute of Technology, Banaras Hindu University, Varanasi 221005, India; 2 Department of Physiology, Institute of Medical Sciences, Banaras Hindu University, Varanasi 221005, India

**Keywords:** antibacterial, bimetallic, gold–silver, multidrug resistance, nanoparticles, wound healing

## Abstract

To date, there are no antimicrobial agents available in the market that have absolute control over the growing threat of bacterial strains. The increase in the production capacity of antibiotics and the growing antibacterial resistance of bacteria have majorly affected a variety of businesses and public health. Bimetallic nanoparticles (NPs) with two separate metals have been found to have stronger antibacterial potential than their monometallic versions. This enhanced antibacterial efficiency of bimetallic nanoparticles is due to the synergistic effect of their participating monometallic counterparts. To distinguish between bacteria and mammals, the existence of diverse metal transport systems and metalloproteins is necessary for the use of bimetallic Au–Ag NPs, just like any other metal NPs. Due to their very low toxicity toward human cells, these bimetallic NPs, particularly gold–silver NPs, might prove to be an effective weapon in the arsenal to beat emerging drug-resistant bacteria. The cellular mechanism of bimetallic nanoparticles for antibacterial activity consists of cell membrane degradation, disturbance in homeostasis, oxidative stress, and the production of reactive oxygen species. The synthesis of bimetallic nanoparticles can be performed by a bottom-up and top-down strategy. The bottom-up technique generally includes sol-gel, chemical vapor deposition, green synthesis, and co-precipitation methods, whereas the top-down technique includes the laser ablation method. This review highlights the key prospects of the cellular mechanism, synthesis process, and antibacterial capabilities against a wide range of bacteria. Additionally, we also discussed the role of Au–Ag NPs in the treatment of multidrug-resistant bacterial infection and wound healing.

## 1. Introduction

Infections caused by microbes have turned out to be a significant source of morbidity and death worldwide [1]. The World Health Organization (WHO) reported that antimicrobial resistance (AMR) is among the top 10 health threats confronting humanity in 2021. According to the most thorough report published in The Lancet, more than 1.2 million people have died due to antibiotic-resistant bacterial infections [2]. If current trends continue, it is estimated that over 10 million people will die each year from drug-resistant diseases by 2050. The misuse and abuse of antimicrobials has mostly caused the development of drug-resistant bacteria. Microbial drug-resistant based infections are a worldwide issue that requires immediate action from health authorities and policymakers to avert avoidable deaths. Nanoparticles (NPs) are commonly employed as an alternative to antibiotics to target microorganisms. Nanomaterials exhibit broad-spectrum antibacterial properties. Noble metal NPs (Cu, Ag, and Au) have been used in food preservatives, medical device coatings, and medical equipment because they have been demonstrated to have powerful and long-lasting antibacterial effects against a wide range of microbes [3]. Recently, because of their unique physical and chemical characteristics, bimetal nanoparticles have been proposed as a viable antibiotic replacement. Bimetallic gold–silver nanoparticles have opened a new field of research due to their broad-spectrum antibacterial capabilities. Because of the synergy between the metals that make up their composition, they have outstanding characteristics and a wide range of uses in the biomedical field [4,5]. Silver nanoparticles have shown the highest surface plasmon resonance (SPR), whereas gold nanoparticles, on the other hand, have great chemical stability. Bimetallic gold–silver nanoparticles demonstrated SPR bands that can be exploited in the phototherapy of cancer and catalytic biosensors [6,7,8]. Recently, AlZaban et al., synthesized bimetallic gold–silver (Au–Ag) core–shell NPs for the catalysis of the trans-esterification process in a fungal isolate (*Fusarium solani)* and to enhance biodiesel production [9]. In another study, Renones et al., investigated the reducing property of Au–Ag NPs loaded with TiO_2,_ that photocatalyzed the conversion of CO_2_ with water [10]. Additionally, Aazam et al., employed bimetallic Au–Ag NPs for the elimination of chromium (VI) as an adsorbent in a liquid solution [11]. The microgeometry and electrical structure of the initial nano-sized single metal were both altered when two distinct metal atoms were combined. This combination caused an impact of synergy that enhanced the catalytic functioning of the new nanoalloy by improving their selectivity, activity, and stability [12,13,14]. To date, the antibacterial properties of different metallic nanoparticles have been studied, and there is consensus regarding silver nanoparticles as them being the most effective against bacteria [15,16,17]. Gao et al., synthesized Ag NPs with diverse morphologies and evaluated their antibacterial properties under controlled settings. Their results revealed that triangular Ag nanoplates have superior antibacterial capabilities than Ag nanospheres [18]. The findings supported that the size and shape of nanoparticles significantly affect their antibacterial properties [19]. However, the exact mechanisms by which silver nanoparticles eradicate pathogens are mostly unknown. It was expected that the Ag^+^ ions are released from the nanoparticles that are mainly responsible for their antibacterial activity. The low stability and reported toxicity of Ag NPs to mammalian cells limit their antibacterial applications [17,18,19,20,21]. Humans generally come into contact with Ag NPs by skin contact, inhalation, oral intake, or blood circulation. Numerous studies reporting on the in vivo and in vitro toxicity of AgNO_3_ are based on their amounts, size, and exposure length to the mammalian cells or tissue [22,23]. In an in vitro study, it was observed that Ag NPs are toxic to mammalian cells originating from the integumentary, hepatic, pulmonary, neurological, and circulatory systems as well as the sexual organs [24]. Similarly, the in vivo toxicity of Ag NPs administered via inhalation, ingestion, or intravenous/IP injection has been reported, and they are detectable in blood and cause harm to several organs, including the lungs, abdominal organs, and nervous system [25]. In a study, Li et al., performed a study on *D. magna*, a freshwater filter-feeding crustacean to test the comparative toxicity of characterized Au, Ag, and bimetallic Ag–Au NPs. The results revealed that all of the nanoparticles examined had dose-dependent toxicological effects on *D. magna.* The LC_50_ of Au was found to be 70 mg/L while Ag had 30 μg/L (Au is approximately 1000-fold less toxic than Ag), whereas for Au–Ag NPs, the LC_50_ value was found to be 12–15 μg/L (depending on the composition of Au and Ag) [26]. Additionally, the liver and lungs are the primary destinations or targets for prolonged Ag NP exposure [27]. To overcome the current restrictions, Ag NPs must be prepared with various compositions to increase their characteristics and suitability for therapeutic applications.

Among all metallic nanoparticles, silver nanoparticles have incomparable antimicrobial potential, while gold nanoparticles (Au-NPs) have good antibacterial activity, high biocompatibility, and the ability to change surfaces in many ways [28,29]. Recently, Ni et al., showed how to quickly and easily make uniform porous hydroxylapatite-decorated Ag nanocomposites that have excellent antibacterial properties against *Escherichia coli* (*E. coli*), *Pseudomonas aeruginosa* (*P. aeruginosa*), and *Staphylococcus aureus* (*S. aureus*) [30]. Similarly, Zhang et al., synthesized polyvinyl alcohol (PVA) nanofibers with embedded silver nanoparticles that were used as antibacterial agents [31].

**Figure 1:**
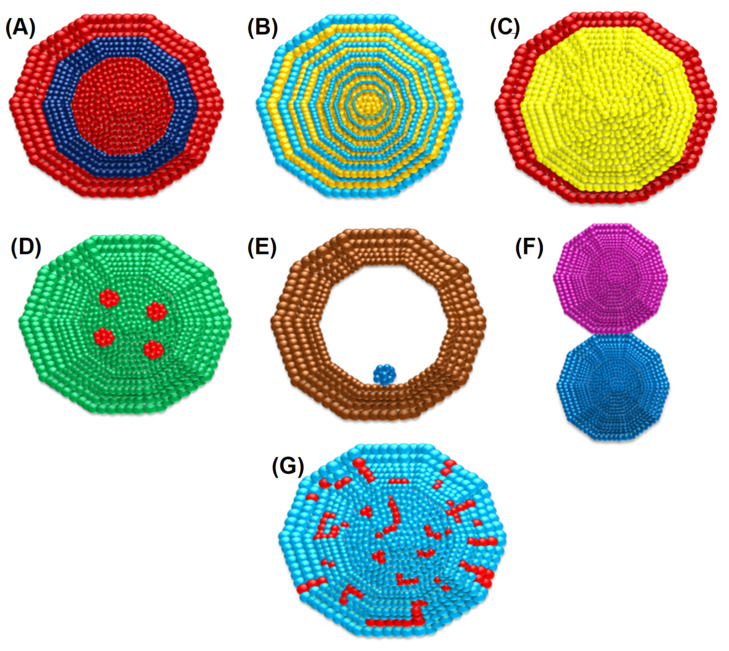
Various arrangements of bimetallic atoms in the nanoparticles. (**A**) Alloy, (**B**) intermetallic, (**C**) subclusters, (**D**) core–shell, (**E**) multishell core–shell, and (**F**) multiple-core materials coated by a single shell and (**G**) disordered alloy. (Different metal atoms are depicted in different colors).

Some small organic molecules (such as specific amines, pharmaceutical intermediates, and indole derivatives) can complement Au NPs and have proved to be effective even against MDR strains [5,32,33]. Many bimetallic NPs (Au–Pd, Au–Pt, and Mn–Fe, etc.) were effective against MDR bacteria [34,35]. In a study, Bahrami et al., reported that the efficacy of antibiotic drugs improved after combining them with an Ag–Au alloy. Bimetallic Au–Ag NPs and their antibacterial effects are based on the biological activities of bimetallic Au–Ag NPs, not on monometallic NPs (Au–NPs or Ag–NPs) [36]. Over the last few decades, many workers have reported the synthesis of bimetallic Au–Ag NPs using diverse techniques [35,37,38,39,40,41]. There are two classes of bimetallic nanostructures: the first are a mixed type and the second are isolated ones. Further, they can be divided into alloys, intermetallic, subclusters, and core–shell based on the configuration of the atoms (Figure 1). It is possible to synthesize bimetallic core–shell NPs made of an Ag/Au core or Au/Ag shell. Mohsin et al., synthesized bimetallic Ag(core)/Au(shell) and Au (core)/Ag (shell) core–shell nanoparticles successfully in the aqueous phase by using the citrate reduction method (changes in salt concentration, the temperature ranged from 25 to 100 °C, and the pH was 2 to 12 of salt solutions for both sets of reaction conditions during the production of the shell structure) [42].

In the current review article, we provided insight into the various synthesis methods for gold–silver NPs. The mechanism of antibacterial activity, the preventive role against multidrug-resistant bacterial infection, and the promotion of wound healing were also covered.

## 2. The Antibacterial Mode of Action of Au–Ag NPs

**Figure 2:**
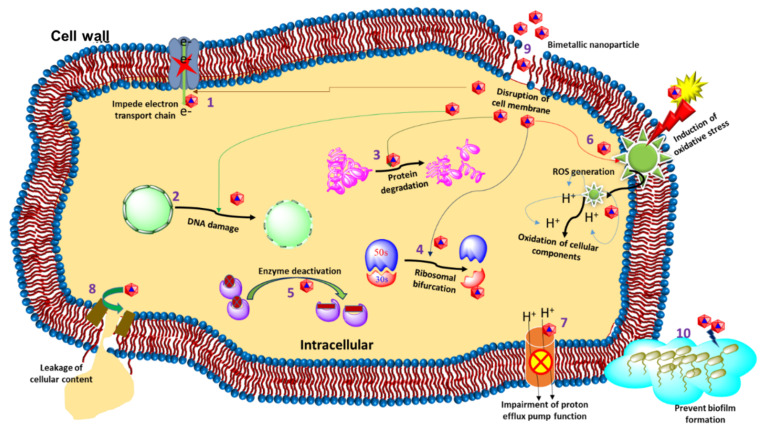
Different antibacterial modes of action displayed by bimetallic Au–Ag NPs. (1) Impede electron transport chain, (2) DNA damage, (3) Protein degradation, (4) Ribosomal bifurcation, (5) Enzyme deactivation, (6) Reduces ROS generation, (7) Impairment of proton efflux pump function, (8) Leakage of cellular content (bacteria), (9) Disruption of the cell membrane and (10) Prevention of biofilm formation.

The invasion of silver ions into the bacterial cell is thought to be the antibacterial action of silver complexes. Silver salts, e.g., AgNO_3_, Na_3_C_6_H_5_O_7_, etc., are not the only source of metal complexes and can affect the target sites [43,44,45]. Bimetallic Au–Ag NPs, just like any other metal NPs, can differentiate bacterial cells from mammalian cells due to the presence of various metal transport systems and metalloproteins. As illustrated in Figure 2, bimetallic Au–Ag NPs are predicted to have antibacterial activity in a variety of ways.

Au–Ag NPs have certain advantages over traditional organic antimicrobials because the target is not limited to the biochemical processes of bacteria (replication, transcription, and translation). However, it also affects other molecular targets, which primarily include the alteration in cell membrane structure due to genotoxicity, protein, and enzyme damage. Additionally, signal transduction inhibition controls bacterial growth due to altered electrostatic interactions, disturbed homeostasis via protein binding, ROS production, oxidative stress, and protein and enzyme damage [46]. The possible mechanism of Au–Ag bimetallic NPs for antibacterial activity is described below:

### 2.1. Cell Membrane Degradation

The bacterial cell surface and spores are negatively charged due to the acid functional groups in proteins (at a physiological pH). Gram-negative bacteria have a more significant negative charge than Gram-positive bacteria, resulting in a higher charge/surface area in the lipopolysaccharide lipid bilayer than other phospholipids [47]. Electrostatic communication begins at the NPs surface when positively charged NPs come into contact with negatively charged bacterial cell membranes. The electrostatic force of attraction increases as the surface area increases. The rise in the ratio of exterior surface area/unit of mass in NPs makes this impact more apparent than in bulk equivalents. The interaction between bacteria and nanoparticles changes the structure and permeability of bacterial cell membranes, leading to oxidative stress and the amplification of bacterial protein damage [48,49].

After cell wall breakage, the water component of cytosol comes out, which causes the cell to discharge its cytosolic components. Cells try to counterbalance this situation through bacterial electron transport systems and proton efflux pumps [50]. The blockage of cellular respiration disrupts vitality transmission, and cell death arises from the ensuing ion shortage and the suppression of membrane stability caused by cell wall cracking [51]. Due to the participation of metal-based NPs, these sorts of occurrences have been seen (silver, gold, magnesium oxide, titanium oxide, and zinc oxide). Metallic NPs, such as silver, have been shown to interact with the sulfurous components of the cell membrane, causing the ion generation to block the cell wall arrangement [52]. Apart from this, metallic NPs can upset cellular respiration and influence cell division, as well as affect DNA synthesis [53].

### 2.2. Disturbance in Homeostasis

The exposure of bacteria to metal or metal ions creates an interruption in the normal metabolic function of bacteria. Metallic NPs’ antibacterial activity is revealed when they attach to cytosolic proteins, DNA, and enzymes. The negative charge on a lipopolysaccharide gets neutralized by positively charged metal ions that make the outer membrane further permeable. Therefore, the buildup of metal ions inside bacterial cells is due to the electrostatic interaction of metallic ions and bacterial cells. The proliferation of bacterial cells is regulated by the disorganized bacterial cell membrane. Upon the internalization of NPs into the bacterial cell, the respiratory and metabolic functions, as well as ATP generation, begin to be disrupted. Metallic ions bind to the peptidoglycan layer’s SH groups, causing the cell wall to break down [54]. In particular, silver obstructs the replication machinery and different cell division stages by binding with cellular respiration and DNA enzymes. Similarly, Au NPs do this by binding with DNA and regulating the genes within the cell [55,56].

### 2.3. Oxidative Stress and the Production of Reactive Oxygen Species (ROS)

ROS (superoxide anions (O_2_), hydroxyl radicals (OH.), hydrogen peroxide (H_2_O_2_), and organic hydroperoxides) are produced when metallic NPs cluster on the bacteria’s outer cell membrane [57]. The produced ROS may prove to be lethal for microbes [58], as they can break down the bioorganic molecules (amino acids, proteins, carbohydrates, lipids, and nucleic acids, which are macronutrients) of the bacteria. ROS production requires the functioning of the redox cycle, functional moieties containing oxygen groups on the nanoparticle surface, and cell–particle interactions. ROS is produced on nanoparticles mostly as a result of changes in surface electrical characteristics and a reduction in molecular size. Superoxide anion (O_2_) production is facilitated by electron donor/acceptor contacts and interactions with molecular oxygen [59,60].

## 3. Synthesis Routes of BIMETALLIC Au–Ag Nanoparticles

**Figure 3:**
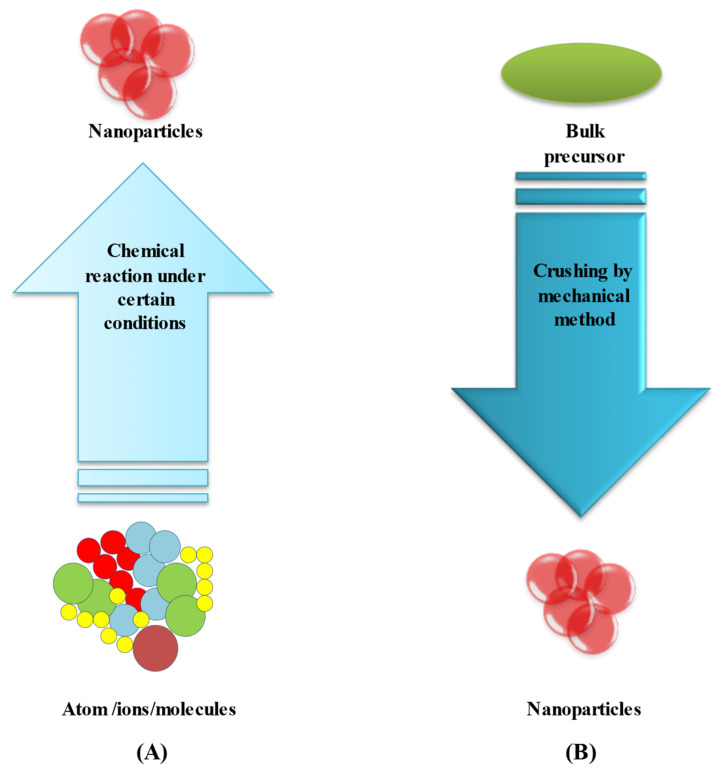
Synthesis methods of nanoparticles: (**A**) bottom-up, (**B**) top-down approaches.

The synthesis and growth mechanism of nanostructures and nanomaterials is one of the most important variables in their use and applications in numerous fields. Although a nanostructured material may have been a promising choice in one application, it may be more useful in another. The technique of growth and synthesis is also important. When it comes to the synthesis of metallic nanoparticles, there are two different methods [61]. The first is known as the top-down strategy, while the second is known as the bottom-up strategy (Figure 3A,B). The top-down technique is useful for producing long-range technical structures and connecting macroscopic devices, and the bottom-up approach is better for producing and arranging short-range orders at the nanoscale. While the former is concerned with shrinking the size of current technological devices, the latter is concerned with the construction of ever more complicated molecular devices on an atomic scale [62]. A detailed description of the synthesis techniques of bimetallic nanoparticles is outlined in detail below:

### 3.1. Bottom-Up Method

The bottom-up method is referred to as the constructive technique. The bottom-up approach is opposed to the top-down approach. Nanomaterials (NMs) of the desired structure, size, and chemical content can be obtained by the growth and self-assembly of atoms and molecules as their building blocks [63]. In a study, Fujimoto et al., synthesized bismuth and platinum (Bi–Pt) bimetallic nanoparticles using a bottom-up technique [64]. Another work was reported by Kawai et al., about the synthesis of bimetallic Au–TiO_2_ (gold–titanium) by the bottom-up technique [65]. The current review mainly focused on the synthesis of bimetallic Au–Ag NPs by various bottom-up methods as discussed below.

#### 3.1.1. Sol–Gel Method

**Figure 4:**
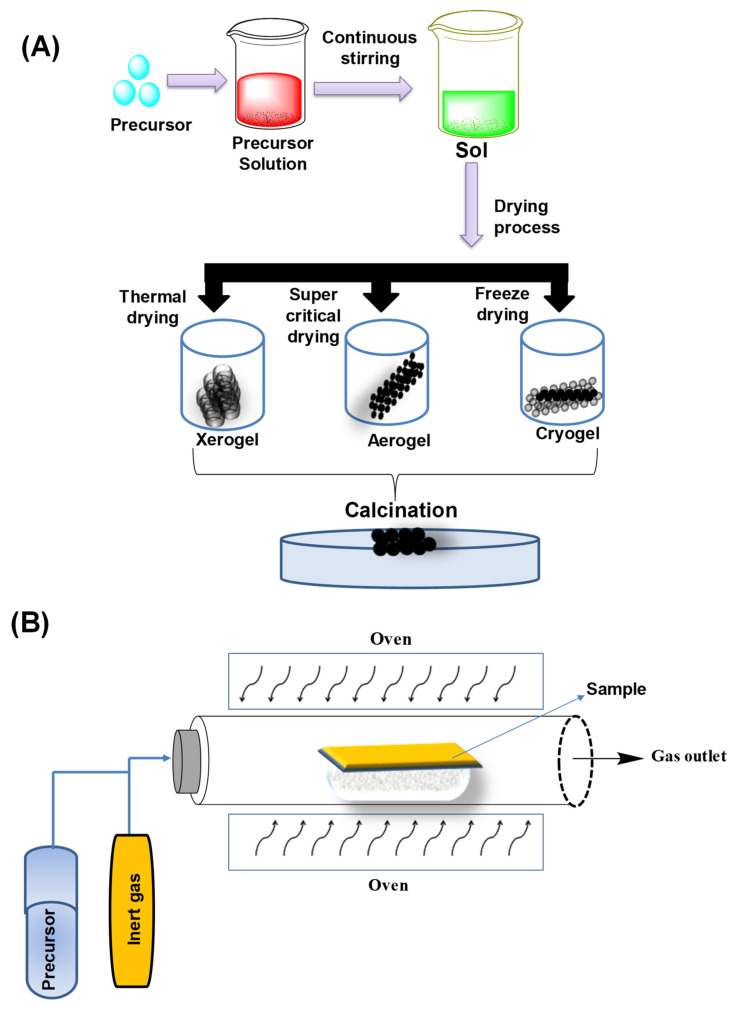
(**A**) Sol–gel method for nanoparticle synthesis. (**B**) Chemical vapor deposition (CVD) method.

This is the most frequent bottom-up technique used for the creation of NMs. Sol is a colloidal solution that consists of solid particles suspended in a liquid. The gel is a solid macromolecule that is dispersed in a continuous medium. The sol–gel technique is a wet-route synthesis method that essentially comprises two reaction pathways. The first is a condensation-derived precursor molecule, and the second is a hydrolysis-derived precursor molecule. This technology has been used to make a wide range of bimetallic NPs with a high degree of homogeneity, including high-quality Au–Ag NPs [66,67]. The sol–gel method for nanoparticle synthesis is presented in Figure 4A.

#### 3.1.2. Chemical Vapor Deposition (CVD) Method

CVD is a versatile technology for nanostructure formation. It has been a popular technique in the microelectronics industry for decades, and it is still one of the most appealing approaches for overcoming the challenges that contemporary technology brings. It is a popular method for making 2D nanoparticles that relies on the production of gaseous reactants (which include the coating element) within a coating chamber. The gaseous species (produced outside) might also be supplied into a coating chamber [68,69]. CVD has already been employed for the creation of bimetallic NPs and is represented in Figure 4B.

CVD offers significant benefits as compared to alternative techniques used for the same purposes. The advantages include a regulated crystal structure, surface morphology, and nanostructure orientations, which are possible by controlling process parameters. The produced nanoparticles are dense and pure. The technique for synthesis is reproducible. For difficult-shaped coatings, the material adhesion and uniformity on the substrate are outstanding [70]. The CVD approach, on the other hand, has certain drawbacks. These drawbacks include the material being moved from the deposited substrates to be evaluated further. In addition to this, the higher manufacturing costs are a drawback. A few of the precursors are sometimes toxic, flammable, explosive, and even expensive. The costs of fabrication are increased by several CVD variants. The usage of substrates is limited due to the high deposition temperature of different CVD variants. Sometimes, the production of toxic gases is a reaction byproduct [71].

#### 3.1.3. Green Synthesis

**Figure 5:**
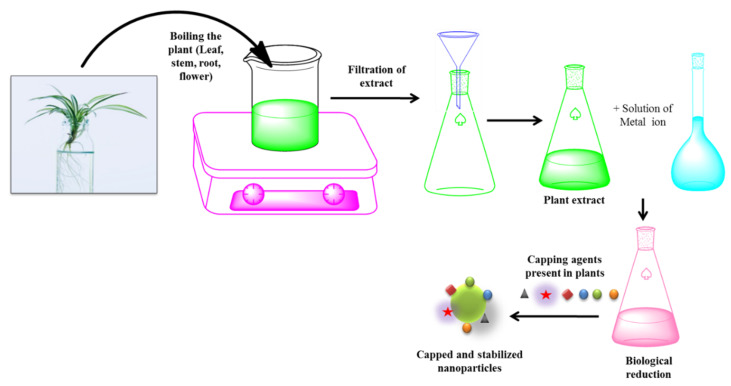
Green synthesis method of metallic nanoparticles.

Various publications are available on the biogenic production of Au–Ag NPs (employing bioactive compounds extracted from various biological extracts) [72,73,74,75] (Figure 5). In a report, Sundarrajan et al., demonstrated the use of *Artocarpus heterophyllus* fruit latex (AHL) for the synthesis of Au–Ag NPs [76]. In phytochemical screening, the crude extract of AHL revealed bioactive components that function as reducing and capping agents in the production of NPs. In another biogenic synthesis method of Au–Ag NPs, algae [77,78] and fungi [79] were utilized to reduce and stabilize Au and Ag, enabling the fast synthesis of stable metallic NPs in a variety of forms sizes, compositions, and mono-dispersities. This method produces Au–Ag bimetallic NPs, which are utilized as antibacterial agents [72,80]. Similarly, Ding et al., synthesized and observed that biogenic bimetallic nanoparticles comprising gold and silver possess significant antimicrobial activity and a lower cytotoxicity than their monometallic counterparts [81,82].

In a recent study, Halder et al., used *Eichhornia crassipes* leaf extract as a reducing and capping agent to create an ecofriendly green approach for the synthesis of Au–Ag bimetallic nanostructures. The Au–Ag NPs were evaluated for their antibacterial activity, and it was found that at a concentration of 100 µM, they efficiently inhibited the growth of *E. coli*. Nieto-Arguello et al., synthesized Ag–Au bimetallic nanoparticles by using starch as a novel coating and reducing agent to span a complete atomic chemical composition range at 10% through a green, highly repeatable, and simple process. Au–Ag NPs were tested for their antibacterial activity against multidrug-resistant *Escherichia coli* (*E. coli)* and methicillin-resistant *S. aureus* (MRSA), and they demonstrated a dose-dependent inhibition even at a 5 µg/mL concentration [83]. A similar study was carried out by Rao et al., to biosynthesize bimetallic Au–Ag core–shell NPs for the first time by using *Ocimum tenuiflorum* (Krishna Tulsi) leaf extract under ambient conditions. The Au–Ag NPs demonstrated a significant bacteriostatic effect against Gram-positive (*B. subtilis*) and Gram-negative (*E. coli*, *P. aeruginosa*) bacteria using a standard antibiotic as a control [84].

#### 3.1.4. Co-Precipitation

**Figure 6:**
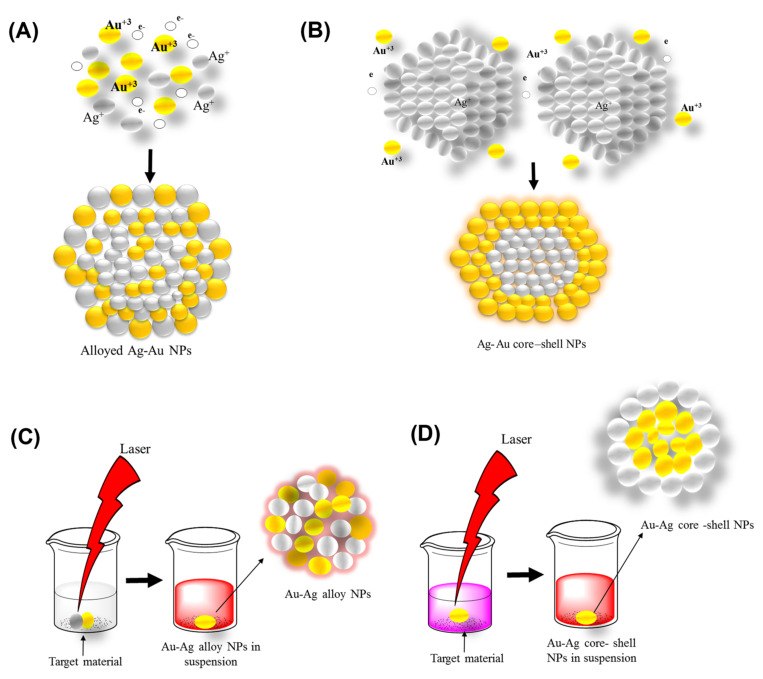
(**A**) Co-reduction method for synthesis of nanomaterials, (**B**) Seeded growth method for Au–Ag NPs synthesis, (**C**) Au–Ag NPs synthesis via simultaneous laser ablation of Ag and Au, (**D**) Au–Ag NPs synthesis by ablation of Ag in Au NP colloid.

The co-precipitation method was earlier used as a wet-chemical procedure to synthesize nanoparticles. This is the simplest and most frequently used method for synthesizing a wide spectrum of NMs (Figure 6A). The capping agent has a prospective role in the stabilization of nanoparticles by preventing nanoparticle aggregations from joining together. These are also crucial in regulating the shape of nanostructures due to their soft-template impact and capacity to alter chemical kinetics. Capping agents have a considerable impact on the directed attachment processes since they directly change the surface of the nanoparticle (the growth of a nanostructure is caused by two processes: Ostwald ripening and oriented attachment). The assembly behaviors of the nanoparticles, which are governed by the balance of forces owing to van der Waals interaction, capillary interaction, surface tension, and hydrophobic attraction, are also greatly influenced by the molecular weight of the capping ligand.

On the other hand, a reducing agent transforms metal ions into nano-metals through the reduction process during the formation of nanoparticles, particularly metal nanoparticles. To accomplish the dual goals of reducing and capping at the same time, some reducing chemicals are utilized in combination with stabilizer molecules. Sometimes (during the production of metal nanoparticles), a reducing agent with a properly designed structure can also serve as the capping agent. Several chemical reducing agents, such as sodium borohydride, sodium citrate, etc., have been used in the synthesis of metal salts into metal nanoparticles.

Other than chemical reducing agents, some natural polysaccharides such as cellulose, starch, heparin, chitosan, dextran, glucose, etc., can be used as reducing agents [85]. Due to the lack of harmful solvents, the production of nanoparticles employing polysaccharides as capping agents is both energy-efficient and entirely environmentally friendly.

In this procedure, impurities precipitate with the product, but they may be readily removed using other processes such as filtration and washing. A precipitation process is used to establish a uniform composition of two or more cations in a homogenous solution in the co-precipitation technique. For the synthesis of bimetallic Au–Ag NPs, this is the most widely applied technique. The slow heating of the reaction component (typically consisting of high-boiling solvents, precursors, and surfactants) is responsible for monomer production, accumulation, nucleation, and growth [86].

In a study, Banerjee et al., developed nanoparticles through the successive reduction of bimetallic Au–Ag alloy nanoclusters with chitosan biopolymer acting as both a stabilizing and reducing agent. Bimetallic Au–Ag alloy nanoclusters were produced [87]. The production of bimetallic Au–Ag NPs, in which Au NPs function as seeds for the continuous deposition of silver atoms on its surface, has been described by Ray et al. and they have also reported the reduction of gold and silver ions in the presence of calixarene in ethanolic solutions, resulting in the production of spherical NPs that are homogenous in size [88]. In a separate study, Kariuki et al., investigated the thin-film assembly of Au–Ag bimetallic nanoparticles through a dicarboxylic acid mediator via interparticle connections at specific metal locations [89]. Additionally, Anandan et al., also reported the sonochemical reduction for the production of bimetallic Au–Ag core–shell NPs by sonicating at room temperature in the presence of alcohol (PEG (0.1%) and ethylene glycol (0.1 M). The core reduction of Au^+3^ metallic gold and Ag^+^ ion metallic silver was completed [90] On the nanometer scale, most metals exist as face-centered cubic structures, which tend to nucleate and develop into twinned and multiply twinned particles (MTPs). Because metal NPs’ intrinsic characteristics are principally governed by their stability, size, and shape, it can be argued that fine-tuning one of these factors is the key to fine-tuning an NP’s attributes [91]. When it comes to managing the size of NPs, the seeded growth strategy has been proven to be an outstanding method. Small metal NPs are generated first and used as seeds (nucleation sites) in this approach, which are then combined with a growth solution to synthesize bigger particles (Figure 6B).

**Figure 7:**
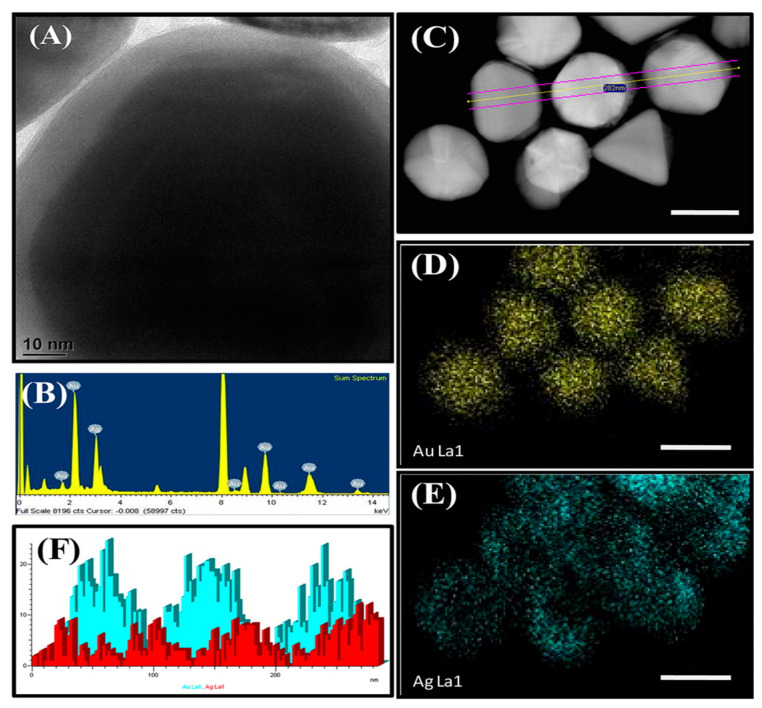
Morphological and elemental characterization of bimetallic (Au–Ag) core–shell nanoparticles and their elemental mapping. (**A**) TEM image, (**B**) EDAX spectra, (**C**) STEM–dark field image, (**D**,**E**) corresponding element mapping images of Au100/Ag bimetallic core–shell NP (scale bar in C–E is 100 nm), and (**F**) EDAX line scan representing Au (cyan) and Ag (red) shown in panel C Reproduced with permission from ref. [92] Figure 3. (ACS Publication^®^ 2014).

In a study, Jia et al., synthesized various combinations of Ag–Au NPs, where gold nanoclusters were used as a seed (prepared by triple helix glucan (lentinan) through microwave-assisted synthesis) [12]. In the bimetallic Au–Ag NPs synthesis, pre-synthesized citrate-capped Au NPs were utilized as the seed. A few years back, Haldar et al., prepared bimetallic core–shell nanoparticles with various core sizes. The core–shell structure of the synthesized nanoparticles was confirmed by the red-shift of the bimetallic plasmonic peaks. The bimetallic nanoparticles showed improved catalytic activity compared to unattached Au and Ag nanoparticles [92] (Figure 7).

Additionally, Berahim et al., synthesized Au–Ag NPs by applying the colloidal seed technique [93]. Co-precipitation methods pose advantages such as easy-to-prepare nanoparticles at low temperatures. Additionally, there are several options for altering the particle surface state and overall homogeneity. However, co-precipitation methods possess some disadvantages as well, which include the fact that the technique will not work if there is a big difference in the precipitation rates of the reactants. Additionally, there is a possibility of the precipitation of contaminants in the product [94].

### 3.2. Top-Down Method

This method is used to transform bulk material into tiny nanoparticles. Top-down techniques are straightforward to employ. However, they are unsuccessful when producing irregularly shaped and very small particles. The main disadvantage of the top-down approach is the difficulty in acquiring a suitable particle size and shape [95,96]. Laser ablation is the most controllable top-down approach. Bulk material is treated with a laser beam (in this case, a bimetallic Au–Ag alloy). Under optimum conditions, well-dispersed bimetallic Au–Ag NPs can be synthesized, which can then be fractionated and surface-functionalized. A two-step synthesis, which involves laser irradiating a combination of silver and gold nanoparticles, is another alternative [97] (Figure 6C,D).

#### Laser Ablation

Laser ablation (LA) is a top-down technique for creating micro/nanostructures by removing molecules from a substrate surface using a laser. It is a green approach to creating metal nanoparticles. Without the use of a surfactant or chemicals, LA is a simple approach to producing metal nanoparticles. Generally, a pulsed laser is used to remove the material from the substrate surface, and it is also possible to ablate the material with a continuous wave laser of a strong intensity. A high-powered laser beam can be used to create NPs by dropping them over a surface of metals, ceramics, glasses, and polymers present in a liquid. The features of nanoparticles created by laser ablation are unique, and no other approach, such as chemical procedures, can duplicate them. The energy, wavelength, laser repetition rate, ablation duration, and aqueous solution absorption are all significant aspects in the production of metal nanoparticles. Ablation occurs when a material absorbs enough energy to evaporate [98]. The LA technique has already been used by many workers to synthesize bimetallic nanoparticles for various biomedical applications. Elsayed et al., used laser ablation to create ZnO–Ag bimetallic nanoparticles with anticancer properties. This is an example of how the LA method is frequently used to create Au–Ag bimetallic nanoparticles [99]. Anugop et al., used laser ablation to make a bimetallic nanocomposite of Au–Ag scattered in chitosan for antibacterial purposes [100].

In a study, Nguyen et al., synthesized Au–Ag NPs using the femtosecond laser reduction method. This approach involves adding an OH scavenger in the form of isopropyl alcohol (IPA) to KAuCl_4_ and AgClO_4_ (precursor solutions). Variation in the precursor ratio permitted the control of the Au–Ag NPs’ composition with an adequate IPA concentration and generated alloy NPs with average diameters below 10 nm with homogenous molar compositions of Au and Ag [101]. Additionally, Alkhayatt et al., reported pulsed laser ablation in the liquid method for the two-step production of bimetallic Au–Ag colloidal nanoparticles [102]. In the first step, Au colloidal NPs were prepared after being laser-targeted onto a piece of high-purity gold plates kept on the bottom of a Pyrex container containing 2 ml of ultrapure deionized water. When pure silver was maintained in freshly produced gold colloidal NPs in the second stage, Au–Ag bimetallic NPs were formed. The antibacterial potential of monometallic gold, silver, and bimetallic Au–Ag NPs (produced by laser ablation utilizing applied pulses (100, 150, and 200) in deionized water) was tested on *S. aureus* and *E. coli* isolates. The inhibitory zone (diameters) revealed that the NPs had synergistic effects on the examined bacteria with diameters of up to 15 mm and 4 mm at the greatest and lowest values, respectively, and this effect increased as the number of laser pulses increased [103]. Heinz et al., reported the synthesis of Au–Ag bimetallic NPs by UV laser irradiation (193 nm wavelength on the glass surface was used in this approach, which was earlier doped with silver ions and then sputter-coated by a thin gold layer) [104]. In a separate study, Abed et al., used pulsed laser ablation in water to synthesize Ag–Au (core–shell) NPs (the laser wavelengths used were 235 nm and 1460 nm) [105].

The ability to produce ligand-free noble NPs in a variety of solvents and with little energy loss are the two main advantages of the laser ablation process. However, laser ablation also possesses a few disadvantages that include this method being highly energy inefficient because it requires a lot of energy to ablate. Additionally, even the most dispersed laser source is almost inefficient in creating NPs on an industrial scale [106].

## 4. Antibacterial Properties of Bimetallic Au–Ag NPs

Controlling the invasion of new bacterial infections, their increasing proliferative powers, and antibacterial resistance, all of which have major public health implications, necessitates the use of extremely potent antimicrobial agents. Due to their synergistic effects, broad spectrum of physiochemical properties, and various mechanisms of action, bimetallic nanoparticles synthesized by combining two distinct metals have recently emerged as having a promising antibacterial efficiency exceeding those of their monometallic counterparts. Consequently, Au–Ag bimetallic nanoparticles are of great importance in imaging, biomedical devices, and nanomedicine [107]. In the current review, numerous recent studies on bimetallic Au–Ag NPs with antibacterial properties were discussed.

In a study, Ding et al., synthesized Au–Ag core–shell NPs via a chemical route and investigated their antimicrobial efficacy. In this study, they reported the aggregation of Au–Ag core–shell NPs onto the bacterial surface, which led to improved imaging because of the improved two-photon photoluminescence. These nanoparticles were found to have antibacterial action against *S. aureus* while being less harmful to human dermal fibroblasts [81]. On the other hand, Bankura et al., reported the use of dextran as a reducing agent for the synthesis of Au–Ag alloy NPs and investigated their antimicrobial efficacy. The antibacterial activity of a 0.1 mg/mL concentration of Ag-Au alloy NPs was found to be significant against bacteria (*B. subtilis*, *B. cereus*, *E. coli*, and *P. aeruginosa*) with zones of inhibition of 24, 21, 17, and 20 mm [108]. In another report, the author followed a photosynthetic route to synthesize Au–Ag alloy nanoparticles for the first time. The bioreduction material in the study was essential oil from *Coleus aromaticus*. Gram-negative *E. coli* and Gram-positive *S. aureus* were used to test the antibacterial efficacy of the photosynthesized Au–Ag alloy nanoparticles. An inhibitory zone of 28 mm for the alloy nanoparticles (synthesized with 150 µL essential oil) demonstrated their strong bactericidal activity against *E. coli*. An in vitro antioxidant assay of the herbal-deduced nanoparticles also exhibited intense free radical (superoxide, hydroxyl, and nitric oxide radicals) scavenging activity [109]. Similarly, Amina et al., prepared Au–Ag alloy nanoparticles by using a microwave-assisted technique that utilized an extract of *Asparagus racemosus* root. In addition, the green-synthesized bimetallic alloy nanoparticles were tested against five different bacterial strains (*Bacillus subtilis* (ATCC 6633), *Escherichia coli* (ATCC 25922), Klebsiella pneumonia (Urine), *Pseudomonas aeruginosa* (ATCC 27853), and *Staphylococcus aureus* (ATCC 25923). It was reported that *P. aeruginosa* and *S. aureus* strains were the most susceptible (highest zone of inhibition) towards Au–Ag alloy nanoparticles versus single metal nanoparticles synthesized with plant extract [110]. Additionally, Gopinath et al., synthesized green bimetallic (Au–Ag) nanoparticles by using *Gloriosa superba* aqueous leaf extract. It was demonstrated that the developed nanoparticles had higher antibacterial as well as antibiofilm activities against Gram-positive and Gram-negative bacteria. The authors found a significant zone of inhibition at 6.33 ± 0.33 mm and 5.33 ± 0.33 mm for *B. subtilis* and *E. coli*, respectively [111]. In Table 1, bimetallic Au–Ag NPs are presented with their associated production methods, inhibition zone, MIC values, and targeted microorganisms.

In another study, recently developed biosynthesized Au–Ag NPs without the incorporation of a surfactant or stabilizing agent. It was observed that when the pH of a solution with *E. coli* and Au ions was raised, Au nanomaterials were formed. Core–shell Au–Ag nanostructures were generated in an ordered manner after Ag ions combined with the Au core. The spectroscopic and microscopic analyses confirmed the structural composition of the biosynthetic bimetallic Au–Ag nanoparticles [121]. In a similar study, Liu et al., reported that their bimetallic NPs showed stronger application possibilities in the superfast colorimetric monitoring of H_2_O_2_, photothermal treatments, and antimicrobial therapy. Without using 3,3′,5,5′-tetramethylbenzidine or peroxidase, their bimetallic Au–Cu NPs were able to sense H_2_O_2_ quickly and calorimetrically [122]. Furthermore, Au–Ag NPs could improve antibacterial activity without increasing cytotoxicity, ensuring that silver could be used in clinical settings [107]. In recent studies, Kalwar et al., created Au–Ag-NP-decorated cellulose nanofibers. Cellulose acetate nanofibers were made by electrospinning, and alkaline hydrolysis was used to deacetylate them. The Au–Ag NPs were coated on the surface of cellulose nanofibers using a dipping process to create an excellent wound dressing material. Furthermore, their antibacterial activity against *E. coli* and *S. aureus* was tested, and the Au–Ag NPs/cellulose was found to be a good antimicrobial material [123].

Villalobos-Noriega et al., synthesized bimetallic core–shell Au–Ag NPs by a green approach. Root extract of *Rumex hymenosepalus* containing catechins and stilbenes acted as a reducing agent in the NPs synthesis. The growth kinetics of microorganisms was analyzed by the Gompertz model. The findings suggested that silver NPs and bimetallic Au–Ag NPs had a dose-dependent effect on the lag phase and growth rate of *E. coli* and *Candida albicans*, with the Au–Ag NPs having a better response [120,124].

## 5. Bimetallic Nanoparticles Targeting Multidrug-Resistant Bacteria

Multidrug-resistant (MDR) bacteria are widely recognized as one of the most serious current public health issues, killing an estimated 700,000 people each year throughout the world [125]. Furthermore, treating MDR bacteria with ineffective antibiotics promotes the expansion of bacterial tolerance. For example, almost more than 50% of *S. aureus* strains obtained from several US hospitals are methicillin-resistant, with some strains also being resistant to vancomycin and carbapenems [126]. MDR microorganisms are frequently linked to nosocomial infection. Some MDR bacteria, on the other hand, have become common sources of community-acquired illnesses. This is a significant breakthrough since community-wide MDR bacteria dissemination leads to a significant rise in the population at risk and causes an increase in the number of MDR-bacteria-related diseases. When the incidence of resistance patterns in bacteria causing community-acquired infections exceeds a certain threshold, broad-spectrum antibacterial and/or combination antibacterial therapy is indicated for the empiric treatment of community-acquired disorders. Efforts to combat drug-resistant diseases are being hampered by the sluggish discovery of new antibiotics. It is anticipated that there will be no effective antibiotics available by 2050 if no new antibiotics are discovered [127]. Due to the lack of effective antibiotics against MDR bacteria, developing nanoparticles has been used as a substitute. It has also been observed that bimetallic NPs are efficient against bacteria, including MDR bacteria [128]. Several studies have shown bimetallic NPs to be effective against MDR bacteria. When monometallic counterparts were joined to form bimetallic NPs, the antibacterial activity was increased [129].

In a study, Wang et al., reported that Au NPs and mercaptophenylboronic acid (MBA) are incapable of acting as antibiotics separately. However, when MBA was coupled with Au NPs, the Au–MBA NPs showed significant antibacterial activity against Gram-positive MDR clinical isolates (e.g., MDR *Staphyloccocus aureus* and MDR *Staphyloccocus epidermidis*) [130]. In a similar study, Zhao and his collaborators synthesized bimetallic NPs by combining two salt solutions in an aqueous phase and reducing them with sodium borohydride. Monometallic NPs were also synthesized by the same method as the corresponding salt for comparison. The antibacterial NPs’ MIC (minimal inhibitory concentration) against *E. coli* and *S. aureus* were determined. Out of the nine different synthesized bimetallic NPs screened, two types of bimetallic NPs, namely the AuRh and AuRu NPs, showed MICs of 7 and 20 µg/mL, respectively, against *E. coli* and *S. aureus*. All of these bimetallic NPs were ineffective against *S. aureus,* with the MIC for *S. aureus* exceeding 128 µg/mL [131]. Kumar and his colleagues developed carbohydrate-coated bimetallic Au–Ag NPs, which were more effective against MDR strains than their monometallic counterparts (i.e., Ag NPs and Au NPs). The Au–Ag NPs were significantly more capable against Gram-negative MDR *E. coli* and *Enterobacter cloacae* than standard antibiotics. An in vivo study also exhibited that bimetallic Au–Ag NPs were almost 11,000 times more effective than Gentamicin at killing MDR MRSA infecting mice skin wounds. The Au–Ag NPs could heal and regenerate the infected wounds faster and without scarring. The in vivo results showed that Au–Ag NPs are an effective antibacterial agent against MDR strains with no adverse side effects [82].

Other forms of Au–Ag bimetallic NPs have been investigated and their antibacterial activity studied, although mostly as coating agents rather than as a delivery method [132].

## 6. Gold, Silver, and Gold–Silver Nanomaterials for Wound Healing

Wound healing is a complex biological process involving a series of cellular and molecular interactions targeted at repairing the injured tissue and restoring its protective function. The wound healing process occurs simultaneously in four different steps: hemostasis, inflammation, proliferation, and remodeling, all of which occur simultaneously. Various medications are now available on the market that can aid with wound healing. For wound healing, drugs that target blood coagulation, inflammatory reactions, platelet function, and cell proliferation are often employed. Glucocorticoids, nonsteroidal anti-inflammatory drugs, and chemotherapeutic agents are examples of these medications [82,133,134].

Bimetallic Au–Ag NPs are attractive candidates for wound dressing integration due to their high antibacterial potential and reduced toxicity profile compared to monometallic silver and gold NPs (Table 2). According to Mârza et al., the antibacterial characteristics of silver can impact the healing process of skin regeneration, and in the meantime, the antibacterial properties of silver can assist an open wound by avoiding bacterial infection [135].

**Figure 8:**
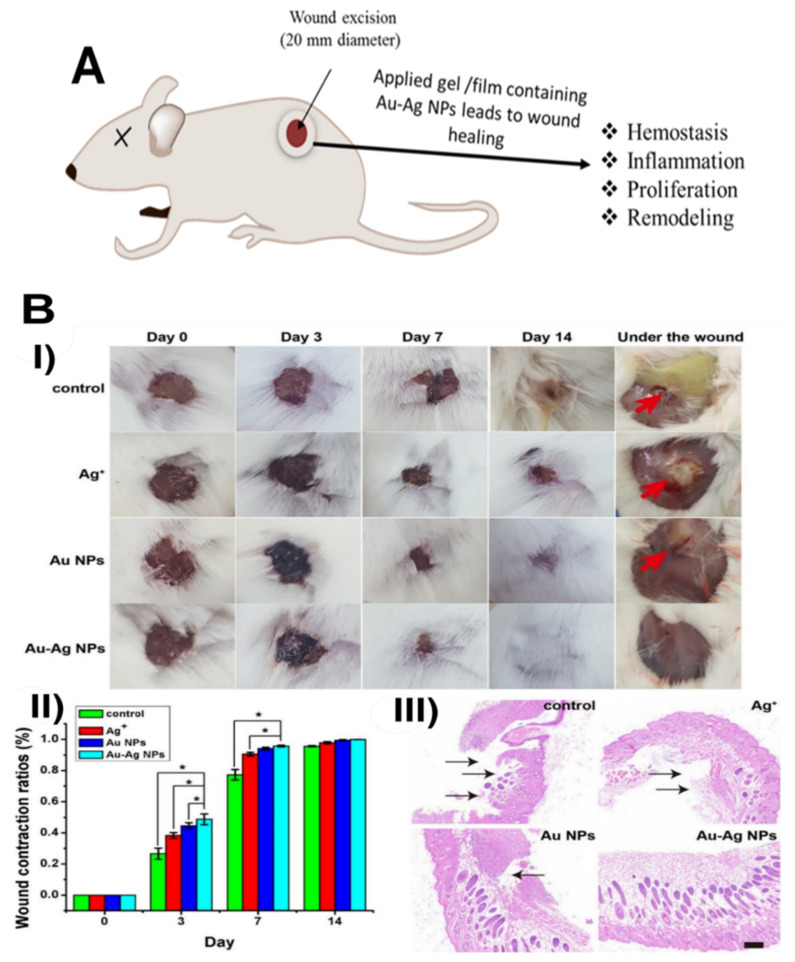
(**A**) Bimetallic Au–Ag NPs showing wound-healing activity in an in vivo rat model. (**B**). (**B**-**I**) Wound-healing activity in the mice infected with *E. coli.* (**B**-**II**) Wound contraction ratios in *E. coli*-infected mice over 0–14 days after treatment with different metallic nanoparticles. (**B**-**III**) Histopathological evaluation of the healed wound after 14 days of treatment with different formulations is presented. In control and monometallic formulation, the wound was not completely healed and damage to the dermis was visible, whereas bimetallic nanoparticles showed completed wound healing without any damage to the epidermis of the skin. * it denotes significant change. Reproduced with permission from ref. [147], Figure 8 (ACS Publication^®^ 2020).

The skin healing and regeneration ability of bioactive glass with spherical gold nanocages in Vaseline ointments were examined in vivo in this study, which compared bioactive-glass–Vaseline and bioactive glass with spherical-gold–Vaseline ointments. Because the spherical gold nanocages were supported by silver, they had a high antibacterial activity. The findings indicated that the presence of silver in a wound affected the healing process. Jiang et al., developed a green synthetic method for bimetallic Au–Ag NPs without using any surfactants and stabilizers. When *E. coli* and Au ions were retained in the same solution, Au nanoparticles were formed first by raising the pH. Core–shell Au–Ag nanoparticles were generated in an ordered manner when the Ag ions combined. Bimetallic Au–Ag NPs showing wound-healing activity in an in vivo rat model has been presented in Figure 8B. [147]. Mârza et al., reported that gold nanoparticles (Au NPs) increased wound healing by promoting tissue regeneration, connective tissue formation, and angiogenesis [136]. Boomi et al., generated phyto-engineered gold nanoparticles from *Acalypha indica* aqueous extract. The wound healing in the animal model indicated that the produced gold nanoparticles had an improved in vivo wound-healing efficacy [137].

## 7. Future Prospects

Organic antimicrobials are more specific, efficient, and highly stable as compared to metal-based nanoparticles. However, metal-based NPs can access modes of action that are generally hard and mostly not possible to attain with organic molecules single-handedly. According to the Community for Open Antimicrobial Drug Discovery, metal-based antibacterial agents displayed a significantly higher hit rate (9.9%) as compared to pure organic antibiotics (0.87%) [148]. It has also been claimed that because of their broad spectrum of molecular targets within a cell, they are less likely to produce microbial resistance [149,150]. It has been reported that bimetallic Au–Ag NPs show excellent cellular interactions with the biomolecules present in bacterial cell surfaces [107]. Bimetallic Au–Ag NPs can also be aimed at a particular target through definite binding activities against selected biomolecules in the bacterial cell. Many workers have used bimetallic Au–Ag NPs for clinical development as therapeutic agents such as anticancer agents and against various infectious diseases.

Unfortunately, so far, no absolute and complete treatment for bacterial resistance is available. Due to a very scarce pipeline of effective antibacterial agents against MDR bacteria, there is an urgent need to investigate the efficacy of bimetallic Au–Ag NPs against various drug-resistant bacteria. On the other hand, to decrease the toxicity of bimetallic Au–Ag NPs, serious efforts are required. The exact biological mechanisms and the reason behind their antibacterial actions are not well established. The relationship between NPs’ size, shape, natural characteristics, and cytotoxicity has not been proposed. For improved outcomes, more research into the use of bimetallic Au–Ag NPs is required. Before widespread biomedical application, there is an ongoing need to clarify the concerned mechanism and the number of toxicities associated with bimetallic Au–Ag NPs [151]. Despite this, their exceptional antibacterial properties and minimal cytotoxicity make them interesting medical nanotechnology players.

## 8. Conclusions

According to recent findings, the manipulating and controlling of materials with dimensions ranging from 1 to 100 nm is now attainable where traditional physics has failed. NMs are rapidly being applied in fields such as physics, material science, chemistry, and healthcare. The top-down (laser ablation) and bottom-up (sol–gel, CVD, biogenic, and co-precipitation) techniques has been successfully used in the synthesis of bimetallic Au–Ag NPs, each with its own set of advantages and disadvantages. Before we can choose the best suitable synthesis method, we must have thorough knowledge of the fundamental mechanics of each synthesis process. Bimetallic Au–Ag NPs have unique physical and chemical characteristics that their monometallic counterparts do not have. The most important use of Au–Ag NPs, regardless of their multiple applications, is as antibacterial agents. However, the toxicological characteristics and pharmacokinetics of Au–Ag NPs should be investigated before comprehensive trends can be established and ultimately predicted. There is currently very little research on the wound healing capability of bimetallic Au–Ag NPs available. As a result, a thorough examination of the molecular mechanisms involving many pathways is required and understanding how these Au–Ag NPs are unique to wound healing will be aided by their involvement. Despite all these limitations, bimetallic Au–Ag NPs are the most promising and emerging competitor in the antibacterial market. In conclusion, the upcoming days are bright for this field and much more promising research on bimetallic Au–Ag-nanoparticle-based antibacterial are expected. However, we are still a long way from achieving the best synthesis methods for Au–Ag bimetallic NPs and defining their precise mode of antibacterial activity, which is a huge research challenge.

## Figures and Tables

**Table 1:** Antimicrobial action of bimetallic Au–Ag NPs.

Material	Method	Microbial Strain	Inhibition Zone (mm) or Rate %	MIC	Other Performance Indicators	References
Au–Ag NPs	Two-photon photoluminescence (2PPL)	*S. aureus*	-	MIC 7.5 ppm against *S. aureus*	Au–Ag NPs showed antibacterial activity by inhibiting biofilm	[81]
Au–Ag alloy NPs	Sodium borohydrate was used for the core reduction of the metal salts	*E. coli* ATCC 25922		-	The antibacterial activity of alloy nano particles was higher than that of pure metals. At a higher molar ratio of silver, the antibacterial activity was improved.	[112]
Au–Ag nanoalloy NPs	Laser ablation	*E. coli* and *S. aureus*			Significant bactericidal	[113]
Au–Ag NPs	Essential oil from *Coleus aromaticus* leaves was used as the reducing agent	*E. coli* and *S. aureus*	*E. coli* with an inhibition zone of 28 mm	*E. coli* with an MIC of NPs with 150 μL essential oil	-	[114]
Ag-Au bimetallic alloy NPs	Microwave-assisted green synthesis of NPs in the presence of *Asparagus racemosus* root extract	*P. eurgnosia* and *S. aureus* strains		-	-	[109]
Au–Ag core–shell NPs	-	*E. coli* and *S. aureus*		-	-	[115]
Au–Ag alloy NPs	-	*E. coli* ATCC strain 25,922 and *S. aureus*		-	-	[116]
Au–Ag core–shell NPs	-	*E. coli* and *S. aureus*		5 µg mL^−1^ for *E. coli* and 7.5 µg mL^−1^ for *S. aureus*	-	[117]
Ag–Au NPs	-	S. aureus				[118]
Ag–Au NPs	Aqueous leaf extract of *Gloriosa superba*	*B. subtilis* ATCC 6633 and *E. coli* (MTCC 40)	6.33 ± 0.33 mm for *B. subtilis* and 5.33 ± 0.33 mm for *E. coli*	-	Antibiofilm activity	[74]
Au–Ag colloidal NPs	Pulsed laser ablation	Gram-positive isolate *(S. aureus)* and Gram-negative isolate *(E. coli)*		-	-	[119]
Au–Ag NPs	On pre-synthesized carboxy methyl tamarind, Au–Ag NPs were synthesized by reducing AgNO_3_ and KAuCl_4_ simultaneously to produce polysaccharide hydrogel-stabilized Au NPs	*E. coli* and *Enterobacter cloacae*		The MIC value of Au–Ag NP for clinical isolate Ec18 was found to be 6 μg/mL and for ATCC MDR isolates BAA-1143 and BAA-2469 was 6 μg/mL	-	[35]
Au–Ag core–shell NPs	Catechins and stilbenes containing root extract of *Rumex hymenosepalus* were used as reducing agents	*E. coli* and *Candida albicans*		*S. aureus* did not grow at a concentration of 100 µg/mL.	-	[120]
Au–Ag NPs	Phytogenic synthesis from a medicinal plant	*M. tuberculosis* and *M. bovis*		Anti TB activity, with MIC of <2.56 μg/mL,		[121]

**Table 2:** In vivo wound-healing activity of gold NPs (Au NPs), silver NPs (Ag NPs), and bimetallic Au–Ag NPs.

Material	Animal Model	Performance Indicator	References
AuNPs			
Bioactive glass (Silicate based) with spherical gold nanocages in vaseline ointment	In vivo study in rats	Bioactive glass with spherical gold nanocages supported by silver had a high antibacterial activity and wound-healing activity.	[135]
Bioactive glass–gold nanoparticles	Experimental rat model	Bioactive glasses led to the promotion of the growth of granulation tissue, while the gold nanoparticles (AuNPs) could induce the acceleration of wound healing by connective tissue formation and angiogenesis.	[136]
Green Au NPs	BALB/c mice	BALB/c mice model with infected diabetic wounds. The re-epithelialization layer was fully covered by green-synthesized Au NPs on the wound area and also collagen filled in the scar tissue when compared with the control group.	[137]
Au–ZnO core–shell	Mice model	Fabrication of Au–ZnO core–shell nanocomposite. Zinc oxide was overlaid on biogenic gold nanoparticles decorated by *Hibiscus Sabdariffa* plant extract.	[138]
Ag NPs			
Ag–ZnO	In Wistar albino rat	Ag–ZnO composite NPs exhibited rapid healing within 10 days when compared with pure Ag NPs and the standard drug dermazin in rat.	[139]
Biosynthesized ZnO/CuO/Ag nanocomposite	Wistar rat	Four metallic NPs were synthesized by biological compounds (*Propolis*). The results strongly suggested the possibility of adopting this unique ZnO/Ag/Ext material dressing compared to other NPs.	[140]
Ag/Ag@AgCl/ZnO hybrid nanostructures (embedded in a hydrogel)	Wistar rat	Ag/Ag@AgCl (hydrogel synthesized via ultraviolet light chemical reduction) followed by the incorporation of ZnO nanostructures by NaOH precipitation). In vivo wound healing showed that Ag^+^ and Zn^2+^ release stimulated the immune system to recruit a high number of WBCs and neutrophils (2–4 times more than the control), resulting in faster wound healing.	[141]
Green Ag NPs	Mice	Green Ag NPs synthesized using *Prosopis juliflora* leaf extract were evaluated for the wound rate of healing activity. The wound closure in the treated mice was much faster than in the other two groups (Carbopol only and Povidone–iodine).	[142]
Green Ag NPs	Rat model	Green-Ag NP-reduction of AgNO_3_ using *Ocimum sanctum*. Skin wound healing was used to test the gel in vivo and the percentage of wound closures was calculated. The gel demonstrated 96% wound-healing activity by the 14th day compared to the standard and control bases.	[143]
Porous silver nanoparticle/chitosan composites	Gelatin-reduced AgNP	Wound healing was accelerated by gelatin/chitosan/Ag, which also had good biocompatibility.	[144]
Chitosan–PVA–silver nanoparticles (CS–Ag NPs)	PVA and chitosan acting as reducing and stabilizing agent	The wound contraction ratio and histological analysis both showed that the CS–Ag NPs promoted wound healing considerably.	[145]
Bimetallic Au–Ag NPs			
Au–Ag NPs	Balb/c mice	A wound (5 mm) was created on the back of mice. The results showed that the Au–Ag NPs were highly effective against MDR-infected wounds in mice with no side effects.	[82]
Silver–gold bimetallic nanoparticles	Silver–gold bimetallic-nanoparticle-decorated nonwoven mats	Showed significant wound healing recovery.	[146]

## Data Availability

Not applicable.
